# Clinical efficacy of thalidomide for various genotypes of beta thalassemia

**DOI:** 10.1186/s12920-024-01963-y

**Published:** 2024-07-18

**Authors:** Wei-jia Yang, Qing-ping Kang, Qian Zhou, Tao Lin, Xiao-min Gong, Cui-juan Huang, Min Dou, Ying Lin

**Affiliations:** 1 Department of Eugenics and Genetics, Guilin People’s Hospital, No. 12 of Wenming Road, Xiangshan District, Guilin, Guangxi Province 541002 China; 2 Department of Hepatobiliary Surgery, Guilin People’s Hospital, Guilin, Guangxi Province 541002 China

**Keywords:** Β-thalassemia, Fetal hemoglobin, Genotype, Thalidomide

## Abstract

**Objective:**

The objective of this study was to investigate the therapeutic efficacy of thalidomide across various genotype presentations of β-thalassemia so as to facilitate the early screening of thalidomide-sensitive thalassemia cases and to understand the impact of iron overload on thalidomide.

**Methods:**

From our initial sample of 52 patients, we observed 48 patients with β-thalassemia for two years after administration of thalidomide. This cohort included 34 patients with transfusion-dependent thalassemia (TDT) and 14 patients with non-transfusion-dependent thalassemia (NTDT). We recorded the values of hemoglobin (Hb), fetal hemoglobin (HbF), and serum ferritin (SF) in the baseline period and at 1, 3, 6, 12, 18, and 24 months after enrollment, as well as the pre- and post-treatment blood transfusion volume in all 48 cases. According to the increase in Hb levels from baseline during the 6-month observation period, the response to thalidomide was divided into four levels: main response (MaR), minor response (MiR), slow response (SLR), and no response (NR). A decrease in serum ferritin levels compared to baseline was considered alleviation of iron overload. We calculated the overall response rate (ORR) as follows: ORR = MaR + MiR + SLR/number of observed cases.

**Results:**

The ORR was 91.7% (44/48 cases), and 72.9% showed MaR (35/48 cases). Among the 34 patients with TDT, 21 patients (61.8%) were free of blood transfusion, and the remaining 13 patients still required blood transfusion, but their total blood transfusion volume reduced by 31.3% when compared to the baseline. We found a total of 33 cases with 10 combinations of advantageous genes, which included 5 cases with βCD41-42/βCD17 and 6 cases with βCD41-42/β-28. Based on the treatment outcomes among the 48 cases in the observation group, there were 33 cases in the MaR group and 15 cases in the SLR/NR group. There was a difference in HbF between the two groups at baseline (*P* = 0.041). There were significant differences between the two groups in Hb and HbF at the time points of 6 and 12 months, respectively (*P* < 0.001). Compared to the baseline measurement, there was a significant decrease in the level of SF at months 12 and 24 (*P* < 0.001).

**Conclusion:**

In this study, we identified 10 β-thalassemia gene combinations that were sensitive to thalidomide. These gene combinations can be used for initial screening and to predict the therapeutic effect of thalidomide in clinical practice. We examined the therapeutic response to thalidomide and found that the administration of thalidomide in combination with standardized iron removal was more beneficial in reducing iron overload.

## Introduction

Thalassemia, a genetic disease, has a high prevalence in the Guangdong and Guangxi provinces of China. Incomplete statistics estimate that there are more than 212 individuals with moderate to severe thalassemia in Guilin City alone. Patients with thalassemia are dependent on blood transfusions and iron removal therapy for prolonged periods, and this imposes a heavy financial burden on their families. Although hematopoietic stem cell transplantation can cure thalassemia, the challenge of finding suitable human leukocyte antigens (HLA) matches and the considerable expenses involved in the transplantation procedure have dissuaded many families from pursuing this option. Additionally, taking or injecting certain medications can have a blood-boosting effect for β-thalassemia, such as HbF activators Hydroxyurea (HU). By activating the γ-gene pathway. It also has the effect of increasing Hb. It typically works well for sickle cell disease but is not significantly effective for most cases of β-thalassemia. Erythropoietin: It stimulates the proliferation and later differentiation and maturation of red blood cells to increase Hb. However, long-term subcutaneous injections are inconvenient, and the potential for Hb improvement is not as significant as with thalidomide. Lenalidomide and pomalidomide: They have shown good effects but are prohibitively expensive for the average working-class family. As these drugs either not significantly increasing Hb, being inconvenient to administer, or being too expensive, they have not been widely used for β-thalassemia.

Thalidomide has been found to effectively improve the symptoms of patients with thalassemia by inducing γ-globin gene expression and stimulating the synthesis of benign fetal hemoglobin (HbF) [1]. Previous studies have shown that the therapeutic outcomes of thalidomide are varied, with some patients remaining free of blood transfusions and others needing reduced blood transfusions [2, 3]. Being affordable and convenient to administer orally, the drug can reduce the financial burden on families and, importantly, save patients a lot of time. It is able to maintain Hb at near-normal levels and also significantly improves the quality of life of the patients.

In our preliminary pilot study, we treated more than 20 cases of β-thalassemia with thalidomide, and 14 of them achieved good therapeutic effects. We also found that certain genotype combinations showed a rapid response to thalidomide; specifically, the levels of Hb and HbF continued to increase even after 1 to 3 months. For instance, in three cases of βCD41-42/β-28 genotype patients, the average hemoglobin (Hb) increased from 84 to 112 g/L, and the average HbF increased from 38.1 to 78.8%. Within 6 months, two cases were transfusion independent. In two cases of βCD17/β-29 genotype patients, after 3 months of medication, the average Hb increased from 91.5 to 124 g/L. However, the effect is not obvious in certain genotypes. For instance, in three cases of βCD17/β-28 genotype patients, the increase in Hb was slow, with the average Hb rising from 81 to 90 g/L within 6 months. Do different genotypes of beta-thalassemia have different responses to thalidomide treatment? There is currently no research on the efficacy of thalidomide in different genotypes of beta-thalassemia. If certain genotypes are found to respond well to thalidomide, it would facilitate rapid case selection based on genotype before clinical use of thalidomide and predict efficacy. In addition, all thalassemia patients require iron chelation therapy.

In the present study, we aimed to explore whether iron overload will affect the efficacy of thalidomide, whether serum ferritin will decrease in patients who have discontinued or reduced transfusions when taking thalidomide, and what are the long-term adverse reactions of thalidomide.

## Materials and methods

### Medical data

#### Guidelines and classifications

We included patients with β-thalassemia who were treated with thalidomide at the People’s Hospital of Guilin City, China, between August 2019 and August 2023. Based on the Diagnostic and Treatment Guidelines for Severe β-thalassemia [4] and the Expert Consensus on the Diagnosis and Treatment of Non-Transfusion-Dependent Thalassemia in Children [5], we classified β-thalassemia into transfusion-dependent thalassemia (TDT) and non-transfusion-dependent thalassemia (NTDT). All patients were informed about the side effects and possible benefits of thalidomide. We required full informed consent from all patients before treatment, and the thalidomide protocol was approved by the Medical Ethics Committee of the People’s Hospital of Guilin City (Approval number: 2021-035KY). The Chinese thalidomide instructions are contraindicated in four situations: (1) Pregnant and lactating women; (2) Children; (3) Allergic individuals; (4) Those engaged in hazardous work, such as drivers and machine operators, due to the side effects of fatigue and drowsiness. In this study, we have formulated a more detailed informed consent form: (1) prohibiting pregnancy during medication for both men and women; (2) timely reporting to the physician if significant fatigue and drowsiness affect learning or work during medication, and necessary reduction or discontinuation of medication if needed; (3) seeking medical attention promptly if significant rash accompanied by itching or other discomfort affects daily life during medication, with dose reduction or discontinuation of medication according to the condition; (3) the good results with thalidomide use in younger ages (some < 10 years old), such as in developing countries like Iran, India, Bangladesh, China, etc., was fully explained to patients and their families before enrollment, and informed consent is signed by guardians for patients < 18 years old. To prevent venous thrombosis, aspirin is taken for those with a baseline platelet count > 500 × 10^9^/L, and blood routine parameters are closely monitored during medication. The following inclusion and exclusion criteria were listed in the informed consent.

#### Inclusion criteria

1. The patient received a clinical and genetic diagnosis indicating homozygosity or compound heterozygosity for β-thalassemia.
2. The age range was 12–55 years, male or female; with an ECOG physical score [6] of 0–2 points.
3. Patients had to provide a written informed consent form that was signed before the commencement of the study.

#### Exclusion criteria

(1) Pregnant and lactating women, as well as participants of reproductive age who were not using contraceptive measures; (2) participants with severe cardiopulmonary diseases, abnormal liver function; and those with serious primary diseases such as cerebrovascular diseases, cardiovascular diseases, liver diseases, kidney diseases, or tumors; (3) those who were allergic to the ingredients of this drug; (4) those who had participated in clinical trials of other drugs within the past month; (5) those who had a history of venous or arterial thrombosis; (6) those who were assessed by the researchers to be unsuitable to participate in this study.

### Treatment schedule

#### Dosage of thalidomide

For patients weighing < 25 kg or aged < 14 years, the thalidomide dose was 37.5 mg/d, and for patients weighing > 25 kg or aged > 14 years, the dose was 50 mg/d. For all patients in the observation group, if there was no response to the medication for six months and the patient requested to stop the medication voluntarily, then the medication was discontinued and the original treatment was resumed; otherwise, the medication was continued until the researchers deemed that there was a situation in which continuation of the medication was not appropriate. During medication, adverse reactions were monitored. Common side effects of thalidomide include fatigue, drowsiness, gastrointestinal reactions, rash, constipation, facial swelling, thrombosis, peripheral neuropathy, and teratogenicity. Patients should be thoroughly informed about these potential side effects before starting treatment, sign an informed consent form, avoid pregnancy, closely monitor their condition, and promptly report any adverse reactions to their physician.

#### Splenectomy

All 20 patients who underwent splenectomy were prescribed aspirin for thrombosis prevention and their platelet counts were closely monitored. Patients were recommended to take 50–100 mg/d of aspirin as a preventive dose, and we monitored the coagulation time every three months if the level of platelets was > 500 × 100^9^/L; if the level of platelets was < 500 × 100^9^/L, the medication was discontinued. Prior to enrollment, all participants in the observational group underwent ultrasound screening for upper and lower limb venous thrombosis. After enrollment, ultrasound screening for deep vein thrombosis of the upper and lower limbs was conducted every 6 months. If a thrombus was detected, medication was immediately discontinued, and the patient was referred to vascular surgery for further evaluation and treatment.

#### Blood transfusion therapy

During the treatment period, patients with Hb < 90 g/L received blood transfusions, and we recorded the pre-transfusion test values.

#### Iron removal therapy

During the trial, all the patients strictly adhered to iron removal therapy, and as per their specific iron removal treatment plan, each patient received a single medication—either deferasirox or deferoxamine. For patients with TDT, we adjusted the dosage based on the Diagnosis and Treatment Guidelines for Severe β-thalassemia (2017 edition) [1]. We monitored serum ferritin (SF) levels every three months, and if the level of SF was < 500–800 ng/ml, iron removal therapy was suspended. Patients with NTDT received treatment as per the Expert Consensus on the Diagnosis and Management of Non-Transfusion-Dependent Thalassemia in Children [2], and if the level of SF was < 300 ng/ml, the iron removal therapy was suspended.

### Detection method

#### Blood routine examination

We used the Sysmex-XE-5000 fully automatic blood cell analyzer to detect whole blood cells.

#### Hemoglobin electrophoresis

For the quantitative detection of hemoglobin components, we used the fully automatic capillary electrophoresis instrument Sebia Capillers 2 flex piercing.

#### Serum ferritin (SF)

We used Roche’s fully automated chemiluminescence immunoassay analyzer, Cobas e801, to detect the levels of serum ferritin.

#### Thalassemia genetype

We used a PCR amplifier (Bio-rad T-100), a medical nucleic acid molecular rapid hybridizer (Yaneng YN-H16), an electrophoresis apparatus (Liuyi, YY-8G electrophoresis), and an AB3500 sequencer to detect four types of deletion-type α-thalassemia: (-α^3.7^/), (-α^4.2^/), (--^SEA^/), and (--^Thai^/). We used multiplex Gap-PCR to detect three types of non-deletion-type α-thalassemia (α^CS^, α^QS^, and α^WS^) with the PCR probe method and identified 18 mutation sites of β-thalassemia common in Chinese patients using the PCR probe method, including CD41-42, CD43, IVS-II-654, IVS-II-28, IVS-II-29, IVS-II-30, IVS-II-32, CD71-72, BE, CD17, CD31, CD37, CD14-15, CD27-28, IVS-I-1, IVS-I-5, CAP + 1 and IntM.

### Observation indicators

#### Parameter collection

We measured the levels of three parameters, namely, Hb, HbF, and SF, over a total of seven times as follows: once at the baseline period and once each at 1 month, 3 months, 6 months, 12 months, 18 months, and 24 months after enrollment.

#### Blood transfusion

We statistically recorded the changes in blood transfusion volume before and after medication. The total transfusion volume (U) for each of the 34 TDT patients in the two years prior to enrollment was recorded. Additionally, the monthly transfusion status and total transfusion volume (U) during the two-year observation period after enrollment for each TDT patient were also recorded.

### Efficacy evaluation

Based on a 6-month observation period, the response to thalidomide was classified into four levels: main response (MaR), characterized by Hb elevation ≥ 2.0 g/L or no need for blood transfusion; minor response (MiR), characterized by Hb elevation of 1.0–2.0 g/L or a decrease in total transfusion volume of ≥ 50%; slow response (SLR), characterized by Hb elevation < 1.0 g/L or a decrease in total transfusion volume between 25 and 50%; and no response (NR) or basically ineffective, characterized by Hb elevation < 1.0 g/L or a decrease in total transfusion volume < 25%. Overall response rate (ORR) = numbers of MaR + MiR + SLR/number of cases observed.

### Statistical analysis

We used SPSS 26.0 to analyze all the data in this study. We used mean ± standard deviation and median to represent the measurement data. Paired t-tests were used to compare the differences in continuous variables pre- and post-treatment, and Pearson correlation analysis was used to analyze the correlation between various factors. A *P* value of < 0.05 was considered to indicate statistical significance.

## Results

### General conditions

**Fig. 1:**
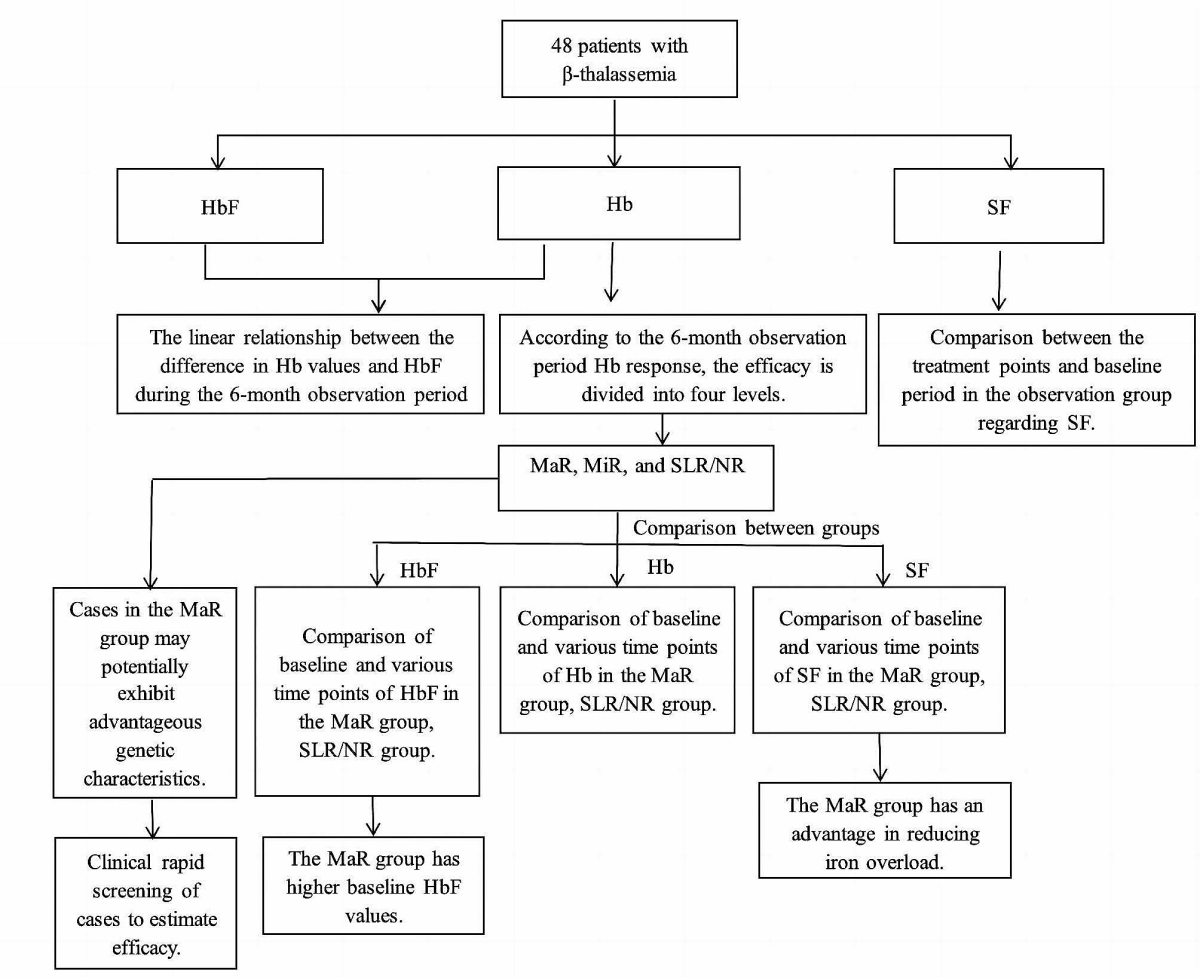
Study flowchart

Forty-eight of the 52 patients completed the 2-year follow-up. Among them, 30 patients were male and 22 were female, ranging from 12 to 41 years old, and the median age was 16 years. Among them, 11 patients (22.9%) were 12–14 years old, 20 were 14–18 years old, and 21 were over 18 years old. With respect to the type of thalassemia, 38 patients were diagnosed with TDT and 14 had NTDT. All NTDT patients had not received any blood transfusion prior to enrollment. Twenty patients out of the 52 had undergone a splenectomy. Four patients with TDT withdrew from the study due to the following reasons: Two of them had a severe rash within two months after taking the drug; one patient developed a menstrual disorder after five months of taking the drug; and one patient changed treatment to undergo hematopoietic stem cell transplantation. Most of the adverse reactions were mild. The most common adverse reactions were mild eyelid/facial edema in 9 cases, followed by rash in 4 cases (2 cases discontinued the drug due to severe rashes, and rash disappeared in the other 2 cases), mild joint swelling and pain of hands and feet in 4 cases, and skin rash in 4 cases, drowsiness in 3 cases, and constipation in 2 cases. The study flowchart is shown in Fig. 1.

In the cohort of 48 patients, we identified 14 distinct combinations of β-thalassemia genes, as delineated in Table 1. Subsequently, patients exhibiting genotypes numbered 1 to 10 (comprising a total of 33 cases) underwent assessment for MaR efficacy, while those with genotypes numbered 11 to 14 (comprising a total of 15 cases) exhibiting SLR or NR efficacy. Based on the observed efficacy, patients were stratified into two groups. Those with genotypes numbered 1 to 10, demonstrating MaR efficacy, were classified into the MaR group, while those with genotypes numbered 11 to 14, exhibiting SLR or NR efficacy, were categorized into the SLR/NR group.

**Table 1:** 48 different gene combinations types of β-thalassemia and statistics on TDT detachment from transfusion

number	gene combinations	NTDT	TDT	detachment from transfusion within 1 m	detachment from transfusion within 1 m	total	effect
①	β-28/β-28	3	0	0	0	3	MaR
②	βCD17/β-29	4	0	0	0	4	MaR
③	βCD41-42/β-28	4	2	2	0	6	MaR
④	βCD41-42/β-29	0	2	2	0	2	MaR
⑤	βCD41-42/βCD17	0	5	5	0	5	MaR
⑥	βCD41-42/βIVS-II-654	0	4	3	1	4	MaR
⑦	βCD17/βCD17	1	2	0	2	3	MaR
⑧	βCD41-42/βCD71-72	0	3	2	1	3	MaR
⑨	βIVS-II-654/βEM	0	2	1	1	2	MaR
⑩	βCD41-42/β-43	0	1	1	0	1	MaR
⑪	βCD17/β-28	2	5	0	0	7	See Table 3
⑫	βCD41-42/βCD41-42	0	4	0	0	4	See Table 3
⑬	βCD17/βIVS-II-654	0	3	0	0	3	See Table 3
⑭	βCD71-72/βEM	0	1	0	0	1	See Table 3
Amount to		14	34	16	5	48	See Table 3

### Efficacy evaluation

#### Changes in Hb in the MaR group

**Fig. 2:**
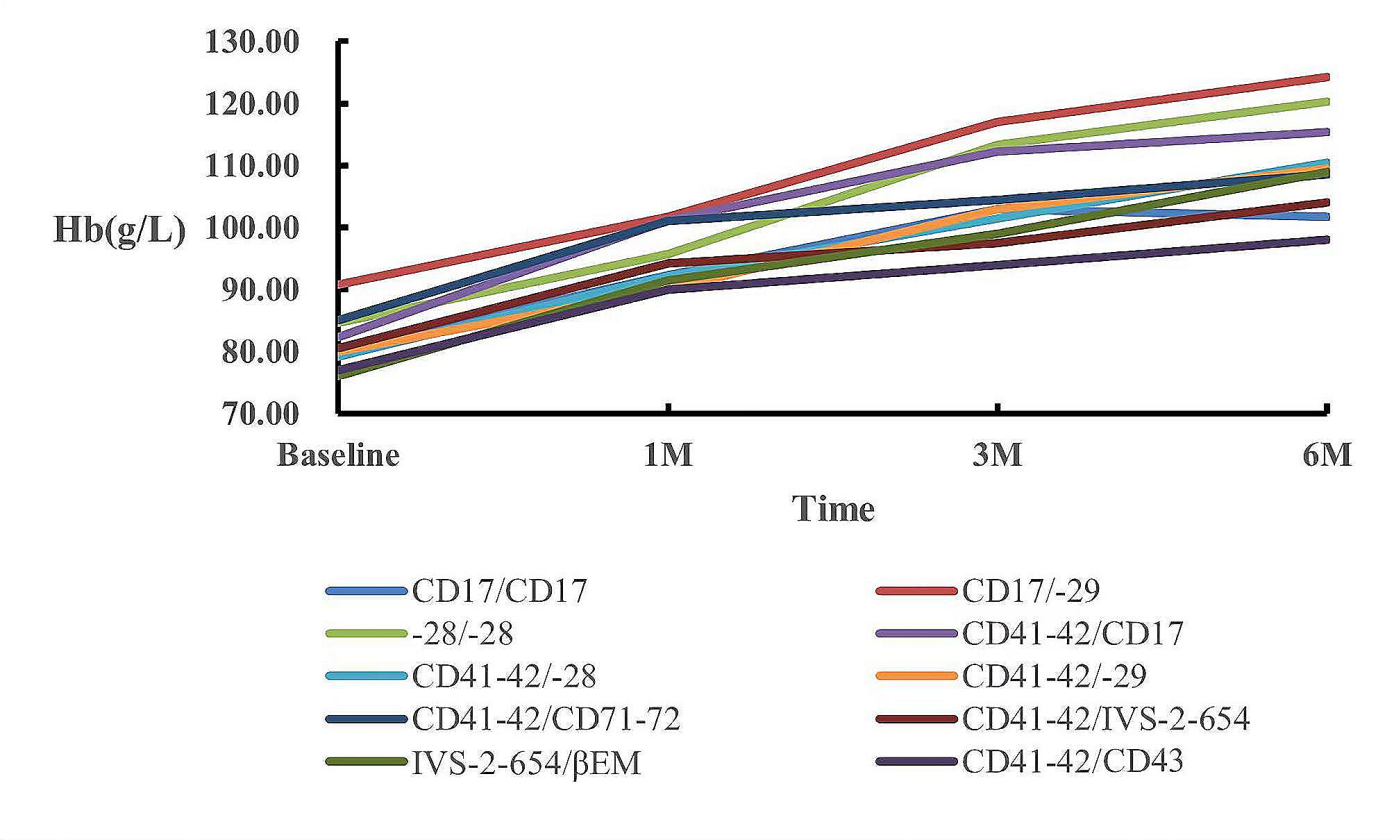
Line chart of Hb changes in the MaR group after 6 M of treatment

Statistics on the increase in Hb in 33 cases after 6 months of treatment are shown in Table 2; Fig. 2. In the analysis, it was observed that 33 cases exhibited an average baseline hemoglobin (Hb) level of 82.15 g/L. After a duration of 3 months (3 M), this cohort demonstrated a mean Hb increase to 106.01 g/L, representing a discernible elevation of 23.86 g/L. Furthermore, at the 6-month mark (6 M), there was a notable average hemoglobin increase of 112.23 g/L.

**Table 2:** Hb values of 33 cases in the MaR group after 6 M of treatment

Gene types		Case	0 M	1 M	3 M	6 M
CD17/CD17	β^0^/β^0^	3	80.33	92.33	103.00	101.67
CD17/-29	β^0^/β^+^	4	90.75	101.75	117.00	124.25
-28/-28	β^+^/β^+^	3	84.67	95.67	113.33	120.33
CD41-42/CD17	β^0^/β^0^	5	82.40	101.20	112.20	115.40
CD41-42/-28	β^0^/β^+^	6	79.17	92.17	101.50	110.50
CD41-42/-29	β^0^/β^+^	2	80.00	90.00	103.00	109.50
CD41-42/CD71-72	β^0^/β^0^	3	85.00	101.00	104.50	108.50
CD41-42/IVS-2-654	β^0^/β^+^	4	80.50	94.25	97.50	104.00
βIVS-2-654/βEM	β^+^/β^+^	2	76.00	91.50	99.00	109.00
βCD41-42/-43	β0/β+	1	77.00	90.00	104.00	122.00

#### Patients achieving transfusion independence

Among the 48 cases, 14 cases with NTDT did not require blood transfusions during the 6-month observation period after the medication, and their response to thalidomide was rated as MaR. They also did not require any blood transfusions during a 2-year follow-up period. Within the cohort of 34 patients diagnosed with TDT, a notable outcome was observed, with 21 patients (61.8%) successfully discontinuing the requirement for blood transfusions. Of these 21 individuals, 16 achieved this cessation within one month of commencing the prescribed medication, while the remaining 5 patients accomplished the cessation at the three-month mark post-medication initiation. The response to thalidomide in these 34 patients was rated as MaR. They also did not require any blood transfusions during a 2-year follow-up period. Among the 48 patients, the response to thalidomide was rated as MaR in 72.9% (35/48 cases). The remaining 13 patients with TDT still required blood transfusions, and the efficacy rating is shown in Table 3. In only 4 cases, the response to thalidomide was ineffective (NR). The total efficacy rate (ORR) of 48 cases was 91.7% (44/48 cases), and the total transfusion volume of 13 patients with TDT decreased by 31.3% when compared to the baseline period.

**Table 3:** Blood transfution of 13 cases TDT and efficacy level

gene types	number		sex	age (year)	Hb(g/L)				reduction in transfusion volume(%)	efficacy level
					baseline	1 M	3 M	6 M		
βCD17/β-28	①	β^0^/β^+^	male	16	66	72	75	79	31.0	SlR
	②		male	12	95	91	87	91	35.2	SlR
	③		female	14	88	95	92	109	41.5	SlR
	④		female	14	77	86	76	103	38.2	SlR
	⑤		male	25	61	64	75	69	34.3	SlR
βCD41-42/ βCD41-42	⑥	β^0^/β^0^	female	12	86	96	94	86	47.7	SlR
	⑦		male	14	76	75	94	86	13.3	NR
	⑧		female	16	54	65	83	84	18.1	NR
	⑨		female	14	61	75	82	83	21.5	NR
βCD71-72/βEM	⑩	β^0^/β^+^	female	16	75	96	89	84	21.4	NR
βCD17/βIVS-II-654	⑪	β^0^/β^+^	female	18	86	90	97	91	30.8	SlR
	⑫		female	12	75	85	65	75	36.9	SlR
	⑬		female	16	78	69	70	75	37.6	SlR

#### Changes of Hb and HbF in the two groups during the treatment cycle

Among the 48 cases, we compared 15 cases in the SLR/NR group and 33 cases in the MaR group for the levels of Hb and HbF at baseline (0 months) and at 6 months, 12 months, and 24 months after the treatment, as shown in Tables 4 and 5. At all four time points, patients in the MaR group had higher Hb and HbF levels than those in the SLR/NR group, and the differences were statistically significant.

**Table 4:** Comparison of hb changes between two groups at baseline and treatment time points

time	group	case	average value	standard deviation	mean value difference	standard error	*P*
Hb0M	SlR/NR	15	74.73	11.49	-7.055	2.662	0.011
	MaR	33	81.79	6.88			
Hb6M	SlR/NR	15	88.67	12.94	-22.576	3.513	< 0.001
	MaR	33	111.24	10.47			
Hb12M	SlR/NR	15	86.47	13.20	-24.018	3.455	< 0.001
	MaR	33	110.49	10.04			
Hb24M	SlR/NR	15	90.2	12.78	-19.133	3.292	< 0.001
	MaR	33	109.33	9.45			

**Table 5:** Comparison of HbF changes between two groups at baseline and treatment time points

time	group	case	average value	standard deviation	mean value difference	standard error	*P*
HbF0M	SlR/NR	15	11.49	11.49	-18.813	7.726	0.019
	MaR	33	30.31	6.88			
HbF6M	SlR/NR	15	35.78	12.94	-46.841	5.313	< 0.001
	MaR	33	82.62	10.47			
HbF12M	SlR/NR	15	39.51	13.20	-47.312	6.062	< 0.001
	MaR	33	86.82	10.04			
HbF24M	SlR/NR	15	41.53	12.78	-46.888	6.035	< 0.001
	MaR	33	88.42	9.45			

There were significant differences in Hb and HbF between the two groups at baseline (*P* = 0.011 and *P* = 0.019, respectively). To mitigate potential confounding factors, a total of 12 cases of NTDT were excluded from the MaR group, while 2 cases of NTDT were excluded from the SLR/NR group. The ensuing comparative analysis focused on 21 cases of TDT categorized as MaR and 13 cases of TDT categorized as SLR/NR during the baseline period (0 months), extending across treatment intervals at 6 months, 12 months, and 24 months. Refer to Tables 6 and 7 for a detailed presentation of the comparative data. There was no significant difference in Hb between the two groups at baseline (*P* = 0.115). However, there was a significant difference in HbF between the two groups (*P* = 0.041). At the treatment time points of 6 months, 12 months, and 24 months, there were significant differences in both Hb and HbF levels between the two groups (*P* < 0.001).

**Table 6:** Comparison of hb changes between two groups of TDT at baseline and treatment time points

time	group	case	average value	standard deviation	mean value difference	standard error	*P*
Hb0M	SlR/NR	13	75.23	12.036	-5.007	3.094	0.115
	MaR	21	80.24	6.01			
Hb6M	SlR/NR	13	85.77	11.05	-22.469	3.672	< 0.001
	MaR	21	108.24	10.00			
Hb12M	SlR/NR	13	84.77	13.39	-22.326	3.97	< 0.001
	MaR	21	107.10	9.74			
Hb24M	SlR/NR	13	86.85	9.54	-19.821	2.786	<0.001
	MaR	21	106.67	6.72			

**Table 7:** Comparison of HbF changes between two groups of TDT at baseline and treatment time points

time	group	case	average value	standard deviation	mean value difference	standard error	*P*
HbF0M	SlR/NR	13	7.14	8.06	-10.957	5.134	0.041
	MaR	21	18.10	17.31			
HbF6M	SlR/NR	13	29.2	19.62	-51.652	5.812	< 0.001
	MaR	21	80.85	14.25			
HbF12M	SlR/NR	13	33.06	28.37	-53.691	7.146	< 0.001
	MaR	21	86.75	13.16			
HbF24M	SlR/NR	13	35.13	27.31	-52.236	7.074	<0.01
	MaR	21	87.37	13.97			

### The relationship between Hb and HbF in the observation group

**Fig. 3:**
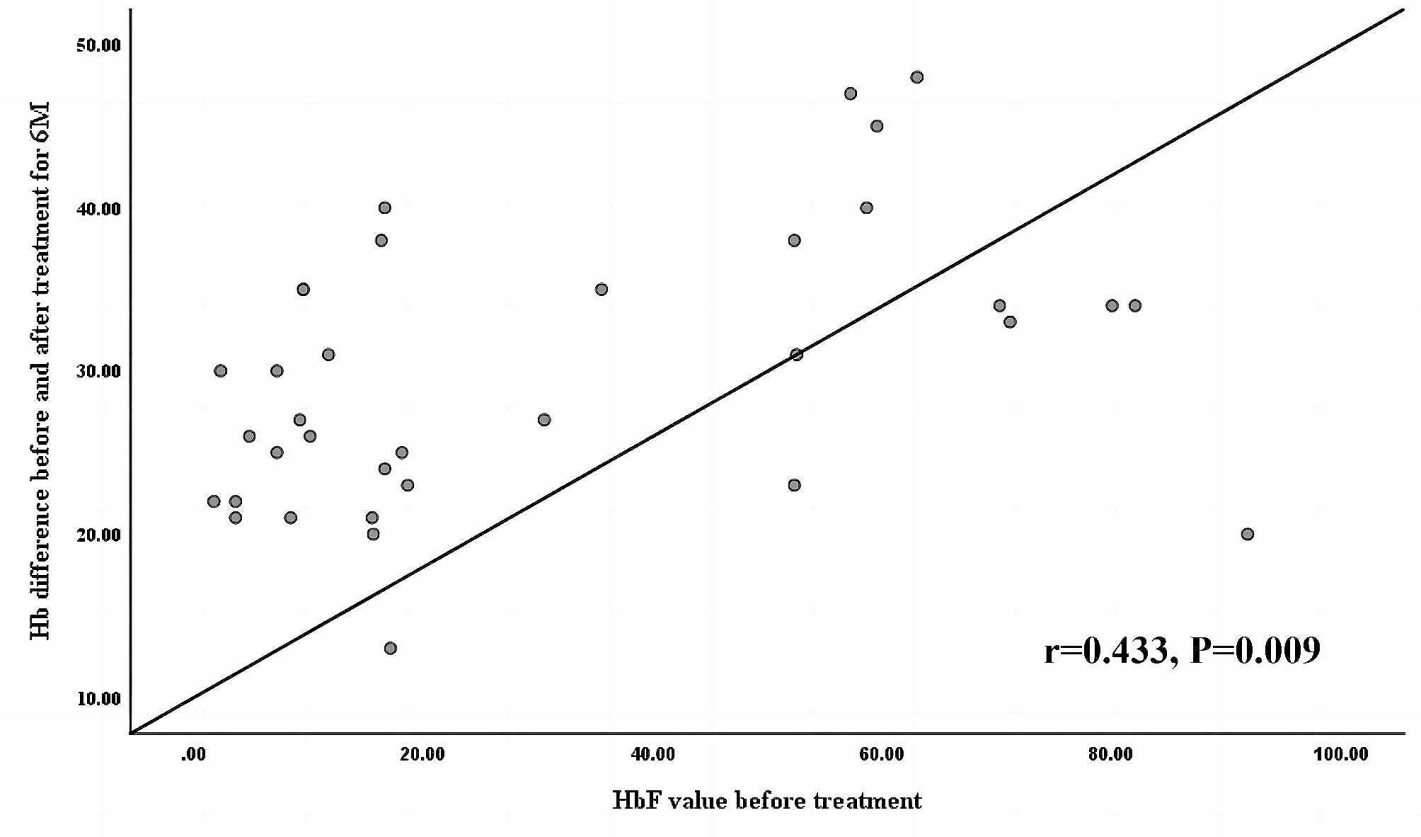
Correlation between Hb difference at 6 M before and after treatment and HbF ratio before treatment

There was a positive linear relationship between Hb and HbF, as shown in Fig. 3.

### Changes before and after chelation treatment

All 48 patients received standardized iron removal therapy. Among them, 4 patients with TDT had to discontinue removal therapy due to low SF levels. The SF values before discontinuation of the therapy are shown in Table 8.

**Table 8:** SF values before and after discontinuation of TDT in 4 cases

sex	age (year)	withtrawal time	before discontinuation medication SF(ng/ml)	24 M SF(ng/ml)	gene types
male	15	6 M	345	345	βCD41-42/βCD17
male	14	12 M	404	346	βCD41-42/βCD17
female	18	18 M	583	336	βCD41-42/βCD17
male	14	12 M	550	459	βCD41-42/βIVS-II-654

The comparison of SF levels between baseline and months 12 and 24 of treatment in the 48 patients is shown in Table 9. There was a significant difference in SF levels between the treatment time points (month 12 and month 24) and the baseline period (*P* < 0.001).

**Table 9:** Changes in SF values over time after treatment in 48 observers

(I)time	(J) time	mean value difference (I-J)	standard error	*P*
0 M	12 M	1490.586	295.779	< 0.001
	24 M	2457.900	445.509	< 0.001
12 M	0 M	-1490.586	295.779	< 0.001
	24 M	967.314	229.968	0.001
24 M	0 M	-2457.900	445.509	< 0.001
	12 M	-967.314	229.968	0.001

The comparison of SF levels of the 21 patients with TDT in the MaR group and the 13 patients with TDT in the SLR/NR group between baseline (0 months) and treatment time points (months 6, 12, and 24) is shown in Table 10. There was no significant difference in SF levels between baseline (0 months) and month 6 (*P* > 0.05), while there was a significant difference in SF levels between month 12 and month 24 (*P* < 0.05).

**Table 10:** Comparison of SF changes between two groups of TDT at baseline and treatment time points

time	group	case	average value	standard deviation	mean value difference	standard error	*P*
SF0M	SlR/NR	13	7137.38	4725.55	2310.885	1414.825	0.112
	MaR	21	4826.5	3509.68			
SF6M	SlR/NR	13	6223	3851.55	2190.762	1169.218	0.07
	MaR	21	4032.24	2943.16			
SF12M	SlR/NR	13	5574	3685.67	2211.143	1040.73	0.041
	MaR	21	3362.86	2400.92			
SF24M	SlR/NR	13	4714.38	3021.29	2285.623	863.701	0.013
	MaR	21	2428.76	2026.50			

## Discussion

### Evaluating the efficacy based on hb levels

The 33 cases in the MaR group showed an average increase in Hb of 30.08 g/L over 6 months, as shown in Table 2. Thalidomide exerts a positive influence on β-thalassemia by enhancing the synthesis of hemoglobin F (HbF) through two main mechanisms: (1) it effectively enhances the expression of GATA-1 and EKLF of hematopoietic progenitor cells, thereby prompting the expression of γ-globin genes [7]; (2) it increases HbF synthesis by activating the p38 mitogen-activated protein kinase (p38 MAPK) signaling pathway through reactive oxidant species (ROS) and acetylizes histone H4, to induce the expression of the γ-bead protein gene [8]. 

In this study, we followed up 48 patients with β-thalassemia (comprising 34 cases of transfusion-dependent thalassemia [TDT] and 14 cases of non-transfusion-dependent thalassemia [NTDT]) for two years. We found that the overall response rate (ORR) of drug treatment was 91.7% (44/48 cases), higher than the rate of 85.7% reported by Ren et al. [9]. for 14 cases of NTDT with a follow-up period of 3 months and higher than the 37 cases (including 14 TDT cases and 23 NTDT cases) reported by Yassin [10] with a median follow-up time of 15 months. This discrepancy might be attributed to the differences in sample sources and varying durations of follow-up.

Of the 34 patients with TDT, 21 (61.8%) achieved transfusion independence and were rated as having achieved the MaR response. This finding was similar to the rate of 63.6% reported in 22 patients with TDT who achieved the MaR in the study by Xiao et al. [11].

### Identification of genomic combinations sensitive to thalidomide

Our study is the first report on the efficacy of thalidomide in different genotypes of β-thalassemia, and we identified 10 combinations of favorable genes that were sensitive to thalidomide. These gene combinations resulted in an increase in Hb levels 1 to 3 months after the treatment, with the highest increase of 29.8 g/L in the βCD41-42/CD17 genotype. All five patients showed clinical manifestations of TDT and achieved transfusion independence after one month of treatment. Of the six patients with the βCD41-42/β-28 genotype, four had NTDT and two had TDT. A month of treatment led to transfusion independence, and Hb rose by an average of 22.33 g/L in the six patients.

The βCD41-42/β-28 genotype showed good sensitivity to thalidomide, as evidenced by the case of a 15-year-old patient with TDT reported by Li [12], who achieved transfusion independence after one month of thalidomide treatment and by the case reported by Ren [13] of a 19-year-old patient with NTDT who experienced an increase in Hb levels from 68 g/L to 113 g/L in three months after thalidomide treatment.

Although patients with the CD41-42/βIVS-II-654 genotype showed an average increase in Hb levels of 16.75 g/L within three months of the treatment, the Hb levels increased to 23.5 g/L after six months. However, as reported by Li [12], three cases with this genotype achieved transfusion independence after six months of treatment, and according to Ren [13], one case with this genotype had an increase in Hb levels from 78 g/L to 103 g/L after three months of treatment. The significant variations in genetic combinations can be attributed to ethnic differences between foreign and Chinese populations, and case reports have indicated the presence of certain favorable genes.

Fozza [14] described the case of a 48-year-old female patient with NTDT with a genotype of βCD39/βCD6 whose Hb levels fluctuated between 60 g/dl and 80 g/dl before treatment, but after one month of medication, her Hb levels rose to 99.9 g/L. After 10 months of medication, her Hb levels were greater than 100 g/L. Another case was a 56-year-old female with βCD39/βCD39 who was unable to undergo blood transfusions due to autoimmune hemolytic anemia. Before treatment, her Hb was 26 g/L and her HbF was 98%. After four years, her Hb remained at 80 g/L and HbF was 98%. As reported by Lilia [15], a 21-year-old female TDT patient with a βCD39/β-28 genotype had an initial Hb level of 46 g/L and an HbF level of 62.3% before treatment. After three months of treatment, the patient’s Hb level increased to 70 g/L, and at the end of the observation period, the Hb level reached 104 g/L with HbF close to 100%.

### The change trend of HbF in the MaR and SLR/NR groups

Researchers [2, 3] have reported that baseline HbF levels can be used as a valuable indicator to predict the efficacy of thalidomide. In our study, we found the baseline HbF level to be positively correlated with the hemoglobin increase after treatment (*P* = 0.009) (Fig. 3), and this finding is consistent with the results reported by Li [12] and Yang [3]. 

We compared 21 cases of TDT in the MaR group and 13 cases of TDT in the SLR/NR group at baseline and treatment time points of 6, 12, and 24 months. There was a significant difference between the two groups in the HbF levels at baseline, with a HbF of 7.14 in the SLR/NR group and 18.10 in the MaR group (*P* = 0.041). There was a significant difference in the changes in HbF between the two groups at each treatment time point of 6, 12, and 24 months, with *P* values < 0.001. Specifically, the HbF was 35.3 in the SLR/NR group and 87.27 in the MaR group at 24 months, indicating a more significant increase in HbF in the MaR group.

There are multiple regulatory factors involved in the control of the γ-globin gene. Fang et al. [16]. reported that thalidomide could induce the expression of the γ-globin gene in human erythroid cells in vitro, leading to an increase in γ-globin production. Downregulation of BCL11A and KLF1 may be one of the mechanisms by which thalidomide induces γ-globin gene expression. Huang et al. [17]. also concluded that transcription factors such as BCL11A, KLF1, and GATA1 may be involved in the process of thalidomide-regulating γ-globin expression.

Simultaneously, at the end of differentiation, thalidomide may slow down the differentiation of the erythroid system by downregulating SOX6 and TAL1, increasing the proliferation of immature red blood cells, and regulating hemoglobin transcription, thereby effectively inducing γ-globin. Zhu et al. [18]. reported elevated levels of miR-223-3P in patients with transfusion-dependent β-thalassemia, and the expression of miR-223-3P decreased after oral administration of thalidomide. Thalidomide can ameliorate anemia by targeting miR-223-3P, indirectly suggesting that downregulation of miR-223-3P levels improves thalidomide efficacy.

The findings of a study by Yang et al. [3]. demonstrated the significant role of HBG2 and HBS1L-MYB gene polymorphisms in the response to thalidomide in patients with NTDT. Patients carrying the polymorphisms at the rs7482144, rs9399137, rs4895440, and rs4895441 sites showed a higher response rate to thalidomide. Categorizing the patients into different groups, they found that individuals with these four polymorphic sites had significantly higher response rates in the MaR group compared to the MiR and NR groups, with statistically significant differences. Additionally, the MaR group had significantly higher baseline fetal hemoglobin (HbF) levels when compared to the minor response (MiR) group and non-response (NR) group (*P* = 0.001). Further research is needed on the mechanisms by which certain gene combinations facilitate MaR therapeutic effects through thalidomide.

### Iron overload and thalidomide

Table 6 shows that the SF values significantly decreased at the 12-month and 24-month time points compared to the baseline (0-month) and the 12-month time points after treatment. Che et al. [19]. reported that after 12 months of treatment, SF levels decreased from 3955.10 ng/ml to 3389.34 ng/ml in 66 patients with TDT (*P* = 0.023). Begum [20], in a study involving 51 cases over a 3-year follow-up in Bangladesh, reported a decrease in SF from 3258.11 ± 2291.91 ng/ml to 2859.65 ± 2072.74 ng/ml (*P* = 0.003). Indian researchers Chandra et al. [21] studied 37 cases of TDT and reported that SF decreased from 1758.9 ng/ml to 1539.6 ng/ml after 6 months of treatment (*P* < 0.001).

In our study, we found that the decrease in ferritin was more significant due to the fact that some patients no longer required blood transfusion and no longer had the iron load caused by blood transfusion. Therefore, we withdrew four patients with TDT from iron removal treatment. A comparison of the serum ferritin (SF) levels of the 21 patients with TDT in the MaR group and the 13 patients with TDT in the SLR/NR group between baseline (0 months) and the treatment time points of 6, 12, and 24 months is presented in Table 10. There were no significant differences in the comparison between at 0 months and 6 months (*P* > 0.05). However, significant differences were observed between the levels at 12 and 24 months (*P* < 0.05). With the prolongation of treatment time, the SF levels in the MaR group could have decreased significantly when compared to the SLR/NR group, possibly due to the reduction of transfusion-related iron overload as a result of transfusion independence.

### Adverse reactions to thalidomide and comparison with other drugs

All patients completed a 2-year follow-up, and overall, adverse reactions were mild. Most of the discomfort that patients reported could be relieved or disappeared over time with continued treatment. Patients could tolerate some symptoms, such as drowsiness and constipation. In this study, 22.9%, that is, nearly a fourth of patients, were less than 14 years of age, indicating that thalidomide can also be used in the younger age group with a recommended biased small dose (25–50 mg/d) and close monitoring during administration of the drug.

Luspatercept is a red blood cell maturation agent with a different mechanism of action from thalidomide. It requires regular subcutaneous injections and has shown promising efficacy in patients with β-thalassemia. Luspatercept targets and binds to specific ligands of the transforming growth factor (TGF)-β superfamily, reducing activation of the Smad2/3 signaling pathway and improving ineffective erythropoiesis, thereby increasing hemoglobin (Hb) levels. It was introduced to the Chinese market in the second half of last year for patients over 18 with β and α thalassemia. Compared to thalidomide, luspatercept has similar efficacy but is over 20 times more expensive and requires inconvenient subcutaneous injections instead of oral administration. Mitapivat is a pyruvate kinase activator typically used to treat pyruvate kinase deficiency and is currently still in the clinical trial phase.

## Conclusion

Through a two-year observation of 48 patients, we discovered 10 potential β-thalassemia genotypes that may affect the efficacy of thalidomide in reducing transfusion dependence or significantly reducing transfusion volume. Clinically, these genotypes can be used for preliminary screening and prediction of thalidomide’s efficacy. Patients who discontinue transfusions or significantly reduce transfusion volume while taking thalidomide should adhere to standardized iron chelation therapy to further reduce iron overload. Thalidomide has mostly mild adverse reactions, is convenient to administer, reduces the financial burden on families, improves learning and work efficiency, and enhances quality of life. In this study, the sample size of 48 cases is considered moderate among all observations of thalidomide treatment for β-thalassemia. Many studies, as cited in our manuscript, had sample sizes of less than 20, with most studies involving a few dozen cases. Therefore, our research has a certain level of representativeness. However, due to the relatively small overall sample size in the study, with 14 genotype variations detected among 48 observers, covering only 8 of the 18 mutation types of β-thalassemia, and some less common mutations not covered, such as 10 mutations not detected at all, and each genotype having few cases, for example, βCD41-42/βCD43 with only 1 case, and βCD17/βEM with only 1 case. Therefore, this study has limitations and further expansion of the sample size is needed to include more genotypes for a more convincing analysis.

## Data Availability

The date will be provided by the corresponding author (Wei-jia Yang) as requested.

## Abbreviations

TDT: transfusion-dependent thalassemia
NTDT: non-transfusion-dependent thalassemia
Hb: hemoglobin
HbF: et al. hemoglobin
SF: serum ferritin
MaR: main response
MiR: minor response
SLR: slow response
NR: no response
ORR: over response rate
HLA: human leukocyte antigen
ECOG: electronics coordinating group
PCR: polymerace chain reaction
GATA-1: globin transcription fator 1 antibody
EKLF: erythroid krupple-like factor
ROS: reactive oxidant species
p38 MAPK: p38 mitogen-activated protein kinase
BCL11A: recombinant B-cell CLL/Lymphoma 11 A
KLF1: krϋppel-like factor 1
SOX6: SRY -box transcripition factor 6
TAL1: t-cell acute Lymphocytic Leukemia
miR-223-3P: microRNA-223-3p
HBG2: homo sapiens hemoglobin, Gamma 2
HBS1L-MYB: HBS1 like translational GTPase-proto-oncogene, transcripition factor
